# 

**Published:** 1872-10-15

**Authors:** 


Money Receipts for September.—Drs. E. S. Cleveland, $6.00; G.W. Lawrence, $5.00; E. W. Edwards, $3.00; W. H. Searls, $3.00; J. M. Steele, $5.00; A. Gaslin, 6.00; L. Asir, $3.00; O. Peck, $6.00; N. Senn, $3.00; J. W. Fink, $3.00; Wm. Law, $3.00; J. H. Stevens, $5.00; Thos. Aiton, $2.00; Chas. Payne, $1.50; W. E. Quine, $5.00; Geo. Dale, $1.50; H. McKennon, $1.50 ; S. D. Mercer, $6.00 ; Martin Clark, $1.50.

